# Mutation screening of the *UBE3A* gene in Chinese Han population with autism

**DOI:** 10.1186/s12888-020-03000-5

**Published:** 2020-12-11

**Authors:** Xue Zhao, Ran Zhang, Shunying Yu

**Affiliations:** Shanghai Mental Health Center, Shanghai Jiao Tong University School of Medicine, 600 Wanping Nan Road, Shanghai, 200030 China

**Keywords:** ASD, 15q11–13, *UBE3A*, HRM, Sequencing

## Abstract

**Background:**

15q11–13 region is one of the most complex chromosomal regions in the human genome. *UBE3A* is an important candidate gene of autism spectrum disorder (ASD), which located at the 15q11–13 region and encodes ubiquitin-protein ligase E3A. Previous studies about *UBE3A* gene and ASD have shown inconsistent results and few studies were performed in Chinese population. This study aimed to detect the genetic mutations of *UBE3A* gene in Chinese Han population with ASD and analyze genetic association between these variants and ASD.

**Methods:**

The samples consisted of 192 patients with autism according to the DSM-IV diagnostic criteria and 192 healthy controls. We searched for mutations at coding sequence (CDS) regions and their adjacent non-coding regions of *UBE3A* gene using the high resolution melting (HRM) and Sanger sequencing methods. We further increased sample size to validate the detected variants using HRM and conducted association analysis between case and control groups.

**Results:**

A known single nucleotide polymorphism (T > C, rs150331504) located at the CDS4 and a known 5 bp insertion/deletion variation (AACTC+/−, rs71127053) located at the intron region of the upstream 288 bp of the CDS2 of *UBE3A* gene were detected using Sanger sequencing method. The ASD samples of case group were 391 for rs71127053, 384 for rs150331504 and 384 healthy controls, which were used to make an association analysis. The results of association analysis suggested that there were no significant difference about the allele and genotype frequencies of rs71127053 and rs150331504 between case and control groups after extending the sample size. Besides, rs150331504 is a synonymous mutation and we compared the secondary structure and minimum free energy (MFE) of mRNA harboring the allele T or C of rs150331504 using RNAfold software. We found that the centroid secondary structure apparently differs along with the polymorphisms of rs150331504 T > C, the results suggested that this variant might change the secondary structure of mRNA of *UBE3A* gene. We did not detect mutations in other coding regions of *UBE3A* gene.

**Conclusions:**

These findings showed that *UBE3A* gene might not be a major disease gene in Chinese ASD cases.

## Background

Autism is a childhood-onset neurodevelopmental disorder characterized by impaired social interactions and communication, restricted interests and repetitive behaviors, the onset of autism is usually before 3 years old. In 2013, the fifth edition of Diagnostic and Statistical Manual of Mental Disorders (DSM-5) removed delayed language development from the list of diagnose criteria, autism covered a host of diseases such as Asperger’s disorder, childhood disintegrative disorder, and pervasive developmental disorder not otherwise specified and redefined itself as autism spectrum disorder (ASD) [1]. Along with changes in diagnosis criteria and improved understanding and awareness about this disease, the prevalence of ASD increased in the past decade according to the report of Centers for Disease Control and Prevention of USA [2]. The worldwide population prevalence is about 1% [3]. The estimated prevalence of ASD in Chinese population ranged from 2.8 to 29.5 per 10,000 persons according to a review that summarized the findings in Chinese population from several areas [4]. Although epidemiological studies have found a number of risk factors for ASD, such as maternal pregnancy and pregnancy complications [5, 6], the cause of ASD is not clear. Multiple twin and family studies have confirmed that genetic factors play an important role in the development of ASD and the heritability is about 90% [7]. Recent genetic studies have found that hundreds of genetic variants, including common and rare variants, contributing to the occurrence of ASD.

15q11-q13 is a very complex chromosomal region and one of the most likely to occur abnormal regions in the genome, which is associated with a variety of neuropsychiatric diseases such as mental retardation and obsessive compulsive disorder [8]. The 15q11-q13 region consists of a large proximal domain (~ 2 Mb) of paternally expressed genes, a smaller maternal expression domain (MED: ~ 500 kb), and a large distal region (~ 2 Mb) of apparently biallelic expression [9]. Individuals with duplications of the 15q11-q13 region caused by inverted duplication of an extra 15q11–13 (dup15) or isodicentric chromosome 15 [idic (15)] show autism features. Moreover, the incidence of maternally inherited 15q11–13 duplications in ASD population is about 1–3%, which is considered the most common copy numbers variants (CNVs) in ASD [10]. *UBE3A* gene is an imprinted, maternally expressed gene which resides on the MED of 15q11–13 region. *UBE3A* gene encodes the E6-AP ubiquitin-protein ligase and is the causative gene in Angelman syndrome (AS), point mutations in *UBE3A* gene were discovered in about 10% cases of AS [11]. AS is also a neurodevelopmental disorder characterized by severe motor and intellectual retardation, ataxia, hypotonia, epilepsy, absence of speech, and characteristic facies. Previous studies have shown that autistic-like features was frequently described in AS patients, this was considered AS comorbid ASD [12, 13]. Overall, *UBE3A* gene was identified as an important candidate gene of ASD.

However, recent studies about *UBE3A* gene and ASD have shown inconsistent results. In the earlier researches, Cook et al. [14] examined multiallelic transmission disequilibrium test (MTDT) on multiple markers of *UBE3A* gene in autistic disorder families, no correlation between these markers and autism was found. Nurmi et al. [15] reported that *UBE3A* gene was associated with autism for the first time, the researchers detected a significant linkage disequilibrium between the D15S122 marker located in *UBE3A* gene and autism in 94 autistic families. Subsequently, Nurmi et al. [9] conducted linkage disequilibrium analysis on multiple single nucleotide polymorphism (SNPs) and genetic markers of *UBE3*A gene in 100 families with autism, but the researchers failed to find evidence for D15S122 marker related to autism. Glessner et al. [16] conducted genome-wide CNVs screening in autism patients and healthy control, found that autism patients carried CNVs in multiple genes of ubiquitin system (*UBE3A*, *PARK2*, *RFWD2* and *FBXO40*). In terms of rare mutation screening, Veenstra-VanderWeele et al. [17] conducted mutation screening in the exons and promoters of *UBE3A* gene in 10 patients with autism, suggested no functional mutations that changed amino acid sequence. Schaaf et al. [18] screened mutations in 21 autism candidate genes including *UBE3A* gene in 339 high-functioning ASD, found non-synonymous mutations in *UBE3A* gene of ASD patients. In the recent studies, Lossifov et al. [19] conducted a whole genome sequencing study in more than 2517 ASD simplex families, found that an autism proband with a T485A missense mutation in *UBE3A* gene. Yi et al. [20] reported that T485A disrupts phosphorylation regulation, hyperactivates UBE3A and increases synapse formation in vivo. In studies of animal model, Nakatani et al. [21] found that the expression level of *Ube3a* gene in matDp/+ mice increased nearly 2 times. To explore the relationship between the dose effect of *Ube3a* gene and autism phenotype, Smith et al. [22] identified that the penetrance rate of autism phenotype in mice carrying *Ube3a* gene triplication was significantly higher than the mice with *Ube3a* gene duplication.

Given that *UBE3A* gene is one important candidate gene of ASD and there was few studies about *UBE3A* gene in Chinese Han people with ASD, we conducted mutation screening for coding region of *UBE3A* gene and aimed to explore the relationship between these mutations and ASD.

## Methods

### Subjects

Firstly, the mutation screening research was conducted on 192 children who were diagnosed as autism and 192 heathy controls subjects called as the primary sample set (the detailed information see Table 3). Then, we further expanded the sample size called as the second set (comprising the primary sample set, 192 cases and 192 controls) (the detailed information see Table 3) for an association study. The patients were fulfilling the criteria for the diagnosis of autism according to the 4th edition of the Diagnostic and Statistical Manual of Mental Disorders (DSM-IV). They were recruited from Shanghai Mental Health Center, Shanghai Jiao Tong University School of Medicine. Patients were excluded if they had known mental and physical illness or chromosomal abnormalities. The healthy controls subjects were volunteers recruited by advertisement from Shanghai Mental Health Center and local community, and brief unstructured interview were evaluated by senior psychiatrists to exclude individuals who suffer from any severe physical diseases, as well as personal and family history of mental diseases. All subjects are Chinese Han descendant. Ethical approval was obtained from the ethics committees of Shanghai Mental Health Center and an informed written consent of participation in the study was signed by the parents or the legal guardians of the studied subjects.

### Gene screening

Five milliliters of the whole blood was taken and ethylenediaminetetraacetic acid (EDTA) was used to be anticoagulant. DNA was isolated according to the established laboratory protocols.

**Fig. 1:**
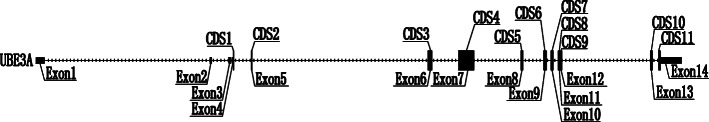
The illustration of Exon1–14 of *UBE3A* gene and CDS1–11

Sequencing data were aligned to the annotated human genome sequence (hg19) using the BLAT tool of the UCSC Genome Browser (http://genome.ucsc.edu/). The *UBE3A* gene sequence was searched from the UCSC Genome Browser database, which showed that there were three spliceosomes (NM_130838, NM_000462 and NM_130839) of *UBE3A* gene. The sequence information of spliceosome 2 includes spliceosome 1 (NM_130838) and 3 (NM_130839). Therefore, the sequence information of spliceosome 2 (NM_000462) was selected as the candidate sequence. *UBE3A* gene has 14 exons (Fig. 1), exon 1, 2, 3, initiation 36 bp of exon 4 and the terminal 1888 bp of exon 14 are non-coding regions (untranslated regions, UTR), the terminal 29 bp of the exon 4, exon 5, 6, 7, 8, 9, 10, 11, 12, 13 and initiation 121 bp of exon 14 are coding sequence (CDS) (Fig. 1). In this study, we performed mutation screening for all coding regions and their adjacent non-coding regions named by CDS1-CDS11, the length of CDS1-CDS11 are 29 bp, 42 bp, 299 bp, 1247 bp, 145 bp, 206 bp, 165 bp, 156 bp, 74 bp, 144 bp, 121 bp, respectively. The total sequenced length after amplifying was 6273 bp (see Tables 1 and 2).

**Table 1:** The primer information for HRM

CDS		Primer sequence	Amplicon length (bp)
3	Forward	TCCCACATGGTTTTCAGGCA	397
	Reverse	GAGAGCTGTACTAATCACTGTGC	
8	Forward	TTTTGCAGACACCTGCTTTCTTA	251
	Reverse	GCAGCCCAATAACTTGTGTTTTGT	
9	Forward	GTCTGAAGCAAAATCACACACCC	256
	Reverse	ATATGTGGAAGCCGGGTAAGAA	
10	Forward	ACGAGGAATGCAAGGTTTTCG	242
	Reverse	ATGAATGCCAAACTGAAACCAGTA	
11	Forward	GTACTGGGACACTATCACCACC	285
	Reverse	TTTCCCATGACTTACAGTTTTCCTG

**Table 2:** The primer information for Sanger Sequencing

CDS		Primer sequence	Amplicon length (bp)
1	Forward	GGTCTTGATTTGAATCGCAGAAA	779
	Reverse	CATTGACACCTAATTTGAAGCTTTG	
2	Forward	ATTTGCTTCTGCATCTTTCACTCT	639
	Reverse	TGTTGTATGGCCACCTGATCT	
4	Forward	TCCATGTGTTCCTATGCTATATGGT	1466
	Reverse	TGAGCCTAGAATGTTTGGCTGT	
5	Forward	CAGTCATGATGTGTGATTCTGGGT	603
	Reverse	TTCCATGTCCTGTGTAGTCCAG	
6	Forward	AGGCACACTCGTTGTAACTACC	800
	Reverse	CCGATGCCACCAAATTACTTACT	
7	Forward	GGGCTTTAGTGCCCAACTGTG	555
	Reverse	GGGACATCACAGTGACTGACAAT	

The two methods high resolution melting (HRM) and Sanger sequencing were performed in our study. The HRM method was used to detect CDS 3, 8, 9, 10 and 11, and the CDS 1,2,4,5,6,7 were directly sequenced by Sanger method according to the amplicon length of every CDS after a series of preliminary experiments. The found variants were further detected in the expanded samples using the HRM method and performed follow-up analyses.

### HRM analysis

The CDS 3, 8, 9, 10 and 11 were amplified using polymerase chain reaction (PCR) and the primers information summarized in Table 1. The primers were designed to generate amplicons of 200–400 bp. Then HRM were performed using LightCycler® 96 Instrument (Roche Diagnostics, Roche Instrument Center AG, Rotkreuz, Switzerland). The amplifications test were performed in 10 μL volumes containing 10 ng of genomic DNA, 2 μmol/L primers, 2.5 mmol/L MgCl2 and 5 μL 2X LightCycler® 96 High Resolution Melting Master (Roche Diagnostics) buffer. PCR cycling included an initial preincubation at 95 °C for 10 min, followed by 45 cycles of 10s at 95 °C, from 65 °C to a “touchdown” at 55 °C, and 30s at 72 °C. The melting program included three steps: denaturation at 95 °C for 10s, renaturation at 65 °C for 1 min, and a subsequent melting cycle consists of a continuous fluorescent reading from 60 °C to 90 °C at a rate of 25 acquisitions per °C. The detected mutations were confirmed with an independent PCR and bidirectional sequencing.

### Sanger sequencing

CDS 1, 2, 4, 5, 6 and 7 were directly sequenced using Sanger method, which could detect about 800 bp sequence length. The length of CDS4 was 1247 bp, which was sequenced forward and backward. The PCR primers information were listed in Table 2. Each PCR master mix included 2 μM of each primer, 1 μl mix dNTP, 1.5 μl MgCl2, 5 μl of 10× PCR buffer, (5 U/μl) AmpliTaq Gold DNA polymerase, 3 μl of DNA sample, and double-distilled water to reach the total volume of 50 μl. PCR condition contained initial denaturation at 95 °C for 5 min, 30 cycles of 95 °C for 30 s and 62 °C–65 °C for 30 s and 72 °C for 45 s, then 72 °C for 5 min as final extension was performed. After purification of PCR products using TaKaRa® Shrimp Alkaline Phosphatase (SAP) and ExonucleaseI (ExonI), sequencing was performed by BigDye Terminator Kit (Applied Biosystems, USA) according to the method by Sanger F, et al. [23]. The sequence information were read using Applied Biosystems (ABI) 3130 Genetic Analyzer (Applied Biosystems, USA). As well, the detected mutations were confirmed with an independent PCR and bidirectional sequencing.

### Association analysis

For each detected variant, we further expanded the sample size of case and control groups, the case-control genotyping studies and association analysis were performed.

The statistic power was calculated using QUANTO software (version 1.2.4.) for allele of the detected variants. The parameters were set to the prevalence of autism to be 1% and a moderate odds ratio (OR) to be 1.3–1.5, the inheritance model was set as log-additive and a type I error rate of 0.05 (two-sided).

Each detected variant were genotyped using HRM method in the expanding samples.

For the association analysis, the online software SHEsis [24] (http://analysis2.bio-x.cn/myAnalysis.php) was used to compare the allelic and genotypic frequencies between the case and control groups. Another online software SNPStats [25] (http://bioinfo.iconcologia.net/snpstats/start.htm) was used to calculate the association between the detected variants and the risk of autism under 5 inheritance models, including codominant, dominant, recessive, overdominant and log-additive models. All of the statistical tests were two-sided, and *p* < 0.05 was defined as statistically significant.

## Results

### Results of mutation screening

HRM method was used to detect mutations of CDS 3, 8, 9, 10, 11 and the results indicated that there were not mutations in CDS 3, 8, 9, 10 and 11 in our tested samples.

The known insertion deletions (AACTC+/−, rs71127053) in the upstream 288 bp of CDS 2 and the synonymous mutation located on CDS4, rs150331504(T>C) were found in the both case and control groups using Sanger sequencing method. The frequencies of rs71127053 in the case and control groups were both 16/192 (0.083), 4/192 (0.02) in the case group and 1/192 (0.005) in the control group carried rs150331504 in our screening samples.

### Results of association analysis

The case subjects were 391 for rs71127053, 384 for rs150331504 and control subjects were both 384 (the detailed information see Table 3) after expanding the sample size in the association analysis. The MAF for rs71127053 and rs150331504 were 0.1276 and 0.0103, and the power to detect a true risk variant in our samples were 42.3–79.8% and 8.5–14% according to OR from 1.3 to 1.5, respectively.

**Table 3:** The information of primary sample set and second sample set

	Primary sample set		Second sample set		
	cases	controls	cases (rs71127053)	cases (150331504)	controls
Total	192	192	391	384	384
Males/Females	154/38	147/45	304/87	299/85	273/111
Mean age ± SD	5.65 ± 3.04	31.59 ± 19.01	5.00 ± 2.99	5.56 ± 2.92	31.49 ± 18.89

*SD* Standard deviation

The results of association analysis for rs71127053 and rs150331504 revealed that there was no significant differences in the distribution of allele (rs71127053: *p* = 0.697, OR = 1.096, 95%CI = 0.691–1.739; rs150331504: *p* = 0.194, OR = 2.013, 95%CI = 0.68–5.918) and genotype (rs71127053: *p* = 0.134; rs150331504: *p* = 0.192) frequencies between the case and control groups (Table S1, see Additional file 1). The genotypes frequencies of rs71127053 showed no significant association with autism under 5 inheritance models (*p* > 0.05) (Table 4).

**Table 4:** Genotype comparison of rs71127053 under different inheritance models

Model	Genotype	OR(95%CI)	*P*-value	AIC
Codominant	1 1 vs 1 2	0.92 (0.54–1.58)	0.31	1001.6
	1 1 vs 2 2	4.83 (0.50–47.07)		
Dominant	1 1 vs 1 2–2 2	1.02 (0.61–1.72)	0.94	1002
Recessive	1 1–1 2 vs2 2	4.87 (0.50–47.39)	0.13	999.7
Overdominant	1 1–2 2 vs1 2	0.91 (0.53–1.56)	0.74	1001.9
Log-additive	–	1.11 (0.69–1.78)	0.66	1001.8

11 represents the wild-type homozygote, 12 represents the mutant heterozygote, and 22 represents the mutant homozygote; *OR* Odds ratio, *CI* Confidence interval

### Bioinformatics analyses

**Fig. 2:**
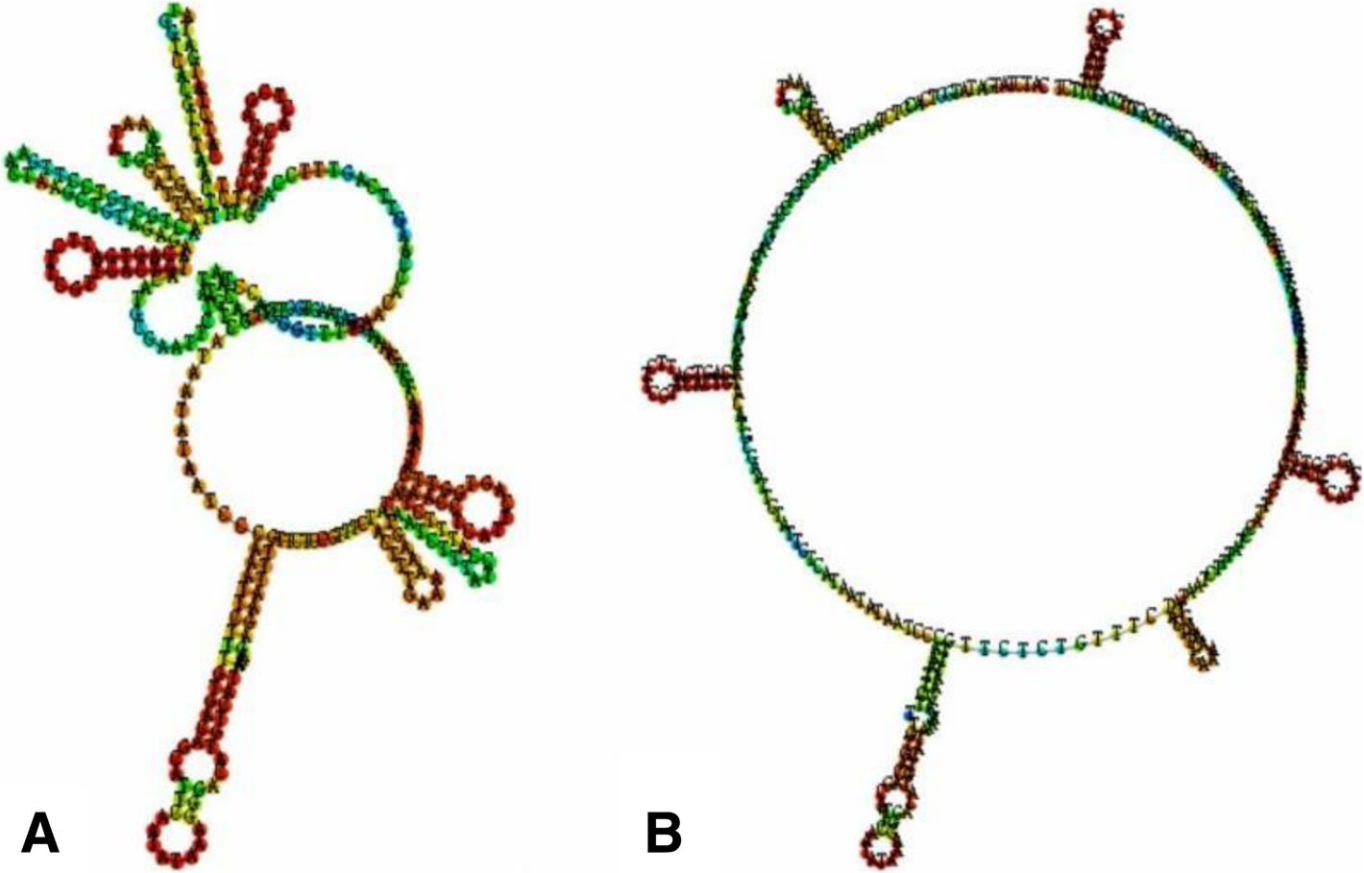
**a** Centroid secondary structure of rs150331504 C allele; **b** Centroid secondary structure of rs150331504 T allele

The 1000 genome project database showed that rs150331504 was a synonymous mutation, which has been shown to be functional by altering the mRNA secondary structure [26]. Although association analysis results showed that there were no significant differences about the allele and genotype distributions of rs150331504 between the case and control groups, the case group carried the mutation frequency had obvious increasing trend. Therefore, the online analysis software RNAfold (http://rna.tbi.univie.ac.at//cgi-bin/RNAfold.cgi/) was used to predict the secondary structure and minimum free energy (MFE) of mRNA harboring the allele T or C of rs150331504. RNAfold prediction result revealed that the centroid secondary structure was markedly changed with rs150331504 T > C alleles (Fig. 2). The MFE of rs150331504 T > C alleles centroid secondary structure was changed from − 47 kcal/mol to − 52.8 kcal/mol.

## Discussion

*UBE3A* gene is a maternal imprinted gene located in 15q11–13, which encoded ubiquitin-protein ligase E3A that played an important role in the ubiquitin pathway. E3A ubiquitin-protein ligase is an important part of the ubiquitin-proteasome system. The ubiquitin-protein ligase consists of 865 amino acids and its C-terminal is a carboxyl-terminal homologous domain (homologous to the E6-AP carboxyl terminus, HECT) of E6-AP. This domain can transfer ubiquitin to the target protein through the ubiquitin-proteasome system to degrade the protein. Ubiquitin is a post-translational modification process that can quickly change the function of the protein, resulting proteasome-mediated degradation of the target protein. With the support of ubiquitin activating enzyme (E1) and ubiquitin binding enzyme (E2), ubiquitin ligase can covalently bind ubiquitin molecules and enzyme substrates. This post-translational modification process can affect a series of biological processes, including protein localization, transport, endocytosis, protein-protein interaction and protein degradation. All these processes depend on the binding of the topological structure of the ubiquitin chain to the substrate protein [27]. In the nervous system, ubiquitin-proteasome system can regulate the function of presynaptic and postsynaptic, including the release of neurotransmitters, the recirculation of synaptic vesicles at the ends of presynaptic, and dynamic change of dendritic spines and postsynaptic density [28]. UBE3A has also been shown to regulate synaptic plasticity, learning and memory formation [29].

Previous studies identified that *UBE3A* gene was an important candidate gene of autism, but there are few studies about the relationship between this gene and ASD in Chinese Han population. Based on the genetic model of common disease rare variants (CDRV), our study investigated the role of *UBE3A* gene variants in the pathogenesis of autism in Chinese Han population. Firstly, 192 patients meeting the diagnostic criteria of DSM-IV about autism and 192 healthy controls were included, *UBE3*A gene was selected as the candidate gene to conduct mutation screening on its coding region and adjacent non-coding regions. We found a known variant rs71127053 (AACTC+/−) on the upstream 288 bp of CDS2. Subsequently, we expanded the sample size and conducted an association analysis. The results revealed that there was no significant difference on the distribution frequencies of allele and genotype of rs71127053 between the case and control groups, rs71127053 did not increase the risk of autism under different inheritance models.

Another known variant rs150331504 (T > C) was detected in the CDS4 region. Similarly, we expanded the sample size and subsequent association analysis showed no significant difference in allele and genotype distribution frequencies of rs150331504 between the case and control groups. The 1000 genome project database showed that rs150331504 was a synonymous mutation. It was generally believed that the non-synonymous mutations can change the amino acid coding sequence, which were related to disease. However, previous studies found that non-synonymous SNPs and synonymous SNPs had the same effect on diseases incidence [30]. Nackley et al. [26] found that synonymous mutations would lead to mRNA structure change, and the most stable structure was associated with the lowest protein level and reduced enzyme activity. In our study, the frequency of rs150331504 was significantly increased in the case group (4/192) compared with the control group (1/192). Considering the role of synonymous mutations in transcription and translation, we compared the secondary structure and MFE of mRNA harboring the allele T or C of rs150331504. We found that the centroid secondary structure apparently differs along with the polymorphisms of rs150331504 T > C, the results suggested that this variant might change the secondary structure of mRNA of *UBE3A* gene and influence its biological functions.

We did not detected any mutations other than the two known variants and the association analysis about the two variants did not find significant difference between ASD cases and controls, which might be due to the following reasons for the negative results. Firstly, we searched the results of genome-wide association analysis (GWAS) for autism in the Psychiatric Genomics Consortium database, no positive sites for *UBE3A* gene were found to be associated with autism at the genome-wide level. These results might suggest that common variants in the *UBE3A* gene were not associated with autism. In addition, inadequate power as a reason of negative findings in genetic association studies must be considered. Though we have expanded the sample size and our sample size is relatively larger compared with the previous association studies about *UBE3A* and ASD, it was still small and difficult to detecting the two variants with relatively low MAF, especially the MAF of rs150331504 was only 0.0103. Although the sample size was one limitation, our study could extend the understanding about the role of *UBE3A* gene in different population and provide some references for future research. Certainly, to achieve a definitive conclusion, the lager study sample size and different ethnic population is imperative. Considering that the role of UBE3A in synaptic plasticity, learning and memory formation and *UBE3A* as a candidate gene for ASD is fairly new, more genetics and mechanisms investigations regarding *UBE3A* gene and ASD should be explored in future. Moreover, we used DnaSP 5.0 software to calculate the nucleotide diversity of the 2628 bp coding region of *UBE3A* gene, and the π value was only 3.8 × 10^− 4^, indicating that the mutation probability of *UBE3A* gene was very low, it is very difficult to detect mutations. At last, previous studies have confirmed that CNVs in 15q11–13 region was related to the risk of autism, indicating that compared with point mutations, structural variants might more easily change the gene function and lead to the occurrence of disease.

HRM and Sanger sequencing were used in our study simultaneously and the two methods was complementary. Currently, HRM has been widely used in SNP typing, mutation screening and epigenetic studies, which is convenient, fast and much cheaper than sequencing technology. Tindall et al. [31] reported that sequences carrying one or more heterozygotes could be effectively distinguished from wild-type by HRM. HRM is widely used in the research of various diseases, especially tumor diseases. In the study of ASD, Kovac et al. [32] used this method to study the three genes SOD1, SOD2 and SOD3 and analyzed the relationship between these genes and ASD successfully. However, HRM also has some limitations. For example, the length of amplicons cannot be too long. Different from sequencing methods, the results of HRM experiment is the melting curve, which needs to be analyzed by software and draw a conclusion, so there would be some deviation in the interpretation of the result. Sequencing technology is the “gold standard” for mutation screening research at present. With the continuous emergence of second-generation sequencing and third-generation sequencing, it has been more and more favored by researchers. However, sequencing technology is very expensive, if HRM and sequencing technology were combined in one study, the total cost will be lower.

## Conclusions

In this study, we investigated the role of *UBE3A* gene in ASD by comprehensive screening for coding sequence regions and their adjacent non-coding regions of *UBE3A* gen for mutations. The results showed that there was no association between the two detected known variants rs150331504 and rs71127053 and ASD. We found that the polymorphisms of rs150331504 T > C might change the secondary structure of mRNA of *UBE3A* gene. We did not detect mutations in other coding regions of *UBE3A* gene. These findings showed that *UBE3A* gene may not play an important role in our ASD cohort.

## Supplementary Information

**Additional file 1: Table S1.** The allele and genotype distributions of rs71127053 and rs150331504 in ASD patients and healthy controls.

## Data Availability

The datasets used and/or analyzed during the current study are available from the corresponding author on reasonable request.

## Abbreviations

ASD: Autism spectrum disorder
CDS: Coding sequence
HRM: High resolution melting
MFE: Minimum free energy
DSM-5: The fifth edition of Diagnostic and Statistical Manual of Mental Disorders
MED: Maternal expression domain
[idic (15)]: Isodicentric chromosome 15
CNVs: Copy number variants
AS: Angelman syndrome
MTDT: Multiallelic transmission disequilibrium test
SNP: Single nucleotide polymorphism
DSM-IV: The 4th edition of the Diagnostic and Statistical Manual of Mental Disorders
EDTA: Ethylenediaminetetraacetic acid
UTR: Untranslated regions
PCR: Polymerase chain reaction
SAP: Shrimp Alkaline Phosphatase
ExonI: ExonucleaseI
SD: Standard deviation
OR: Odds ratio
CI: Confidence interval
HECT: Homologous to the E6-AP carboxyl terminus
CDRV: Common disease rare variant
GWAS: Genome-wide association analysis
